# Study protocol of a cluster-randomised controlled trial assessing a multimodal machine-based exercise training programme in senior care facilities over 6 months – the bestform study (best function of range of motion)

**DOI:** 10.1186/s12877-023-04176-7

**Published:** 2023-08-22

**Authors:** M. Siegrist, N. Schaller, M. Weiß, J. Isaak, V. Schmid, E. Köppel, M. Weichenberger, E. Mende, B. Haller, M. Halle

**Affiliations:** 1 Department of Prevention and Sports Medicine, School of Medicine, University Hospital “Klinikum rechts der Isar”, Technical University of Munich, Georg-Brauchle-Ring 56, Munich, 80992 Germany; 2 Institute of AI and Informatics in Medicine, University Hospital “Klinikum rechts der Isar”, Technical University of Munich, Munich, Germany; 3 DZHK (German Centre for Cardiovascular Research), Partner Site Munich Heart Alliance, Munich, Germany

**Keywords:** Frailty, Sarcopenia, Resistance training, Retirement homes, Ageing, Falls, Exercise

## Abstract

**Background:**

Physical functioning is a crucial factor for independence and quality of life in old age. The aim of the "bestform—Best function of range of motion" trial is to investigate the effects of a 6 months multimodal machine-based strength, coordination and endurance training on physical function, risk of falls and health parameters in older adults.

**Methods:**

Bestform is a cluster-randomised trial including older adults  ≥ 65 years living in senior care facilities in Southern Germany. Senior care facilities are randomly allocated to the control group with usual care (*n* ≥ 10 care facilities) and to the intervention group (*n* ≥ 10 care facilities), overall including  ≥ 400 seniors. Residents belonging to the intervention group are offered a supervised machine-based exercise training programme twice weekly over 45–60 min over six months in small groups, while those in the usual care facilities will not receive active intervention. The primary outcome is the change in Short Physical Performance Battery over six months between groups. Secondary outcomes are change in risk of falling, fear of falling, number of falls and fall-related injuries, physical exercise capacity, handgrip strength, body composition, cardiac function, blood parameters, quality of life, risk of sarcopenia, activities of daily living, and cognition over three and six months.

**Discussion:**

The bestform study investigates the change in physical function between seniors performing exercise intervention versus usual care over six months. The results of the study will contribute to the development of effective physical activity concepts in senior care facilities.

**Trial registration:**

ClinicalTrials.gov: NCT04207307. Registered December 2019.

**Supplementary Information:**

The online version contains supplementary material available at 10.1186/s12877-023-04176-7.

## Background

Due to the demographic change, the ageing society faces individual and common health challenges. On the one hand, life expectancy has increased significantly in Germany and most European countries in recent decades [1]. On the other hand, today’s inactive lifestyle is leading to a significant increase in lifestyle diseases [2] such as hypertension, coronary heart disease or type 2 diabetes and raises the risk of needing long-term care [3] in old age.

Physical inactivity is common in older adults, especially when living in senior care facilities [4], and is responsible for a deterioration in physical performance. One example for impaired physical performance is a reduction of gait speed, which is a determinant to be able to perform daily tasks and to maintain independent [5–7]. Moreover, reduced activity and subsequently reduced muscle strength is also associated with geriatric syndromes such as sarcopenia [8] and frailty [9], increased osteoporosis, risk of falls and increased fracture rates. About 30% of individuals over the age of 65 years report at least one fall per year [10]. Falls increase the fear of repetitive falls leading to a vicious circle of reduced mobility, decline in physical performance and further increase of risk of falls [11].

It has been shown that the participation in effective, multimodal exercise programmes with strength, coordination and balance training can counteract this vicious cycle and can improve physical function, mental well-being, quality of life, and reduce the risk of falling in older adults with or without comorbidities [12–18]. Adaptation of muscle strength by training has even been shown in 70–82 year-old individuals [14]. Therefore, detailed recommendations for exercise interventions are available for older adults [19–21].

However, larger randomised-controlled trials involving very old individuals beyond 80 years applying structured exercises are rare. Particularly machine-based exercises have not extensively been investigated in this patient cohort on a large scale. Moreover, the setting of senior care facilities seems to be ideal for introducing exercise interventions. On the basis of our pilot study (bestform-F; 77 residents; 74–103 years, 85.6 ± 6.6 years; 78% women) that has shown feasibility of a multimodal machine-based strength, balance and endurance training, [22] we have designed the “Bestform-trial – Best Function of Range of Motion”; hereinafter referred to as “bestform”, to examine the efficacy of this training on physical function and risk of falls as well as assessing safety of the intervention. The training programme is performed over a 6-month period in at least 20 senior care facilities in a cluster-randomized setting. The primary endpoint is the Short Physical Performance Battery (SPPB) after 6 months.

## Methods/design

### Design and participants

The prospective cluster-randomised, controlled, two-armed study is organised and conducted by the Department of Prevention and Sports Medicine (Technical University of Munich, Germany). The participating senior care facilities (≥ 10 intervention senior care facilities,  ≥ 10 control senior care facilities) are randomly allocated to one of the two study groups. The participants of the intervention senior care facilities are provided with a physical training programme (“bestform-training”). The participants of the control senior care facilities will not receive any training intervention (usual care), but are provided with information meetings/brochures on a healthy lifestyle. In each intervention senior care facility a machine-based exercise training equipment is installed after randomisation.

**Fig. 1:**
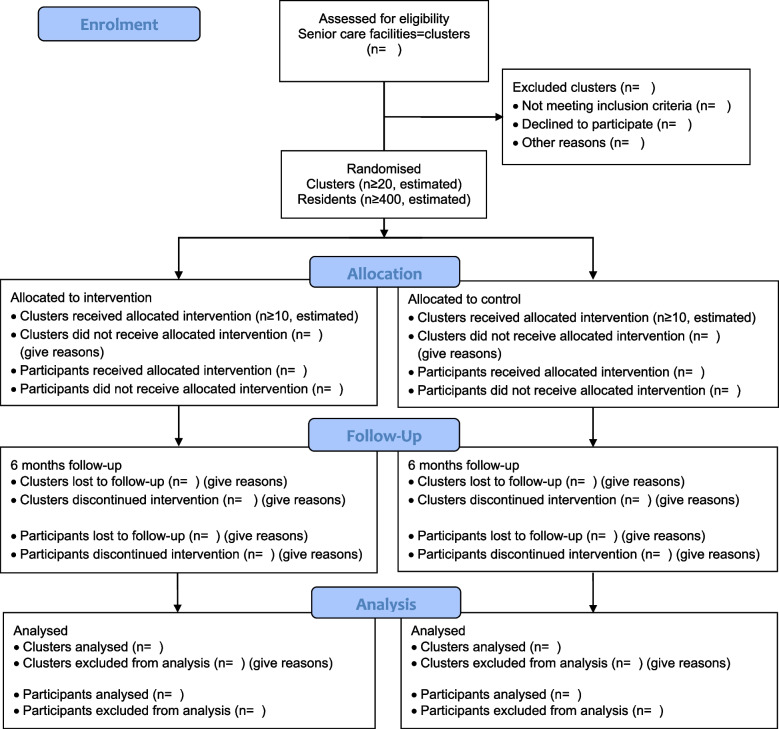
CONSORT Flow diagram showing the bestform-study design and the flow of the participants

The hypothesis of the bestform-trial is that a 6 months multimodal exercise training (machine-based strength, coordination and endurance training) is superior to usual care regarding Short Physical Performance Battery (SPPB) in residents aged ≥ 65 years living in senior care facilities. Secondary endpoints are outlined in Table 2 and obtained on site at three different time points: at baseline, after three months, and after six months. Afterwards, follow-up data will be collected after 18 and 30 months by questionnaires. The study flowchart is illustrated in Fig. 1. Our study protocol is in line with the SPIRIT 2013 statement for clinical trial protocols [23] and subsequent trial reporting considers the CONSORT recommendations for clinical trial reporting [24].

The study has been approved by the local ethics committee of the University Hospital “Klinikum rechts der Isar”, Technical University of Munich, Germany (548/19 S-SR). The study is registered under ClinicalTrials.gov (NCT04207307).

### Recruitment of senior care facilities

Senior care facilities are recruited in the Munich area. A local media campaign and personal telephone calls to senior care facilities will draw attention to the study. A total of 20 senior care facilities is projected, but if the aimed number of participants of  ≥ 400 is not reached, then further facilities will be included until this number is met. Eligibility criteria for senior care facilities include adequate space for implementing exercise facilities and commitment of the general manager of the senior care facility to take part in the study. Study sites include senior homes, offering assisted living as well as caring areas covering the whole spectrum of level of care. Senior care facilities are advised that participation into the trial does not automatically imply participation into the intervention arm. Aside, senior care facilities should preferably have  ≥ 100 residents and an expected participation rate  ≥ 20 residents. Furthermore, no regular machine-based strength training should have been installed. However, care facilities should have the space to set up a fitness facility for a machine-based exercise training programme.

### Allocation of senior care facilities

Participating senior care facilities are allocated randomly to one of the two study groups based on a computer-generated list. Permuted block technique is used to generate group assignments (1:1). The list is stored at the Institute of AI and Informatics in Medicine (AIIM) of the University Hospital “Klinikum rechts der Isar”, Munich, Germany (former Institute for Medical Informatics, Statistics and Epidemiology, IMedIS). Group allocation is performed based on the randomisation list. Individuals involved in the recruitment process and in communication with the senior residences do not have access to the randomisation list.

Allocated senior care facilities are informed whether they have been assigned to intervention or usual care. Thereafter, in intervention senior care facilities, equipment of machine-based exercise training is installed.

### Study participants

Members permanently living in selected homes are informed and invited to participate in the study by an information meeting or personal communication by staff. In case of general participation agreement to be assigned to usual care or intervention, individuals will be informed about the planned investigations including the potential training intervention. To participate in the study, a written informed consent is mandatory to be signed by the study participant or her/his legal representative.

Thereafter, medical history and physical examinations are performed on site (examination 1) in all individuals to assess for inclusion and exclusion criteria. For inclusion, study participants need to be able to stand independently without assistance and be physically and mentally able to participate in the study based on the judgement of the physician performing the clinical examination. Exclusion criteria comprise any acute or chronic illness or physical/mental condition as well as any dementia disease, which does not allow to stand independently or which does not allow physical training in small groups or which would require a training under supervision. All participants have to pass the medical examination including ECG and echocardiography. For this a 12-lead resting electrocardiography (ECG) is conducted to exclude pathologic conditions. This will be added by an echocardiography (ECHO) (CX50, Philips, Netherlands/USA) to assess pathologies e.g. impaired myocardial function or valvular heart disease that pose contraindications for exercise training. Moreover, blood is drawn for analysis of cardio-metabolic risk factors (e.g. blood lipids, blood glucose), safety parameters (e.g. electrolytes, creatinine), parameters of inflammation and acute infection (e.g. high sensitive C-reactive protein) as well as brain natriuretic peptide (pro-BNP) for heart failure screening. Blood samples are stored for future analyses.

### Intervention

The multimodal bestform-intervention consists of a machine-based resistance, coordination and endurance training, which is performed twice per week for 45–60 min in intervention senior care facilities. Age and disability adapted pneumatic resistance training machines (HUR Health and Fitness Equipment, HUR, Finland) are used, including easy access devices for wheelchair users, targeting large muscle groups (leg extension/curl rehab, leg press rehab, push up/pull down EA, chest press EA, optimal rhomb EA).

The exercise is conducted under supervision of qualified exercise instructors (sports scientists, fitness trainers with additional qualifications) and trained undergraduate assistants in small groups of 4 to 6 participants face-to-face in the intervention care facilities.

Prior to intervention start, the participants become familiarized with the exercise training within two introductory sessions (one per week). There, participants are introduced in handling of the machines and the correct execution of the exercises. Also, the entry level of the exercise loads is determined according to the individual´s condition.

Following, the bestform-training (two sessions per week) is carried out over six months and is structured into three phases: 1.) Accommodation phase, 2.) Regular exercise training and 3.) Intensified exercise training (Table 1). During these phases, exercise is increased in duration and intensity on an individual basis. For adaptation the BORG-Scale [25] is used corresponding to percent of 1-Repetition-Maximum (1-RM) [26].

**Table 1:** Overview of the multimodal bestform-training intervention

	**Accommodation phase**		**Regular exercise training**		**Intensified exercise training**	
**Month**	1	2	3	4	5	6
**Duration**	45–60 min	45–60 min	45–60 min	45–60 min	45–60 min	45–60 min
**Frequency**	2x/week	2x/week	2x/week	2x/week	2x/week	2x/week
**Resistance training**						
Repetitions	20	20	15	15	10	10
Sets	2	2	2	3	3	3
Intensity	low (< 60% of 1-RM; RPE < 11)	moderate (60–70% of 1-RM; RPE 11–13)	moderate to high (70–80% of 1-RM; RPE 14–16)	moderate to high (70–80% of 1-RM; RPE 14–16)	high; until muscle fatigue (>80% of 1-RM; RPE > 16)	high; until muscle fatigue (>80% of 1-RM; RPE > 16)
**Coordination / Balance training**						
Dynamic (number of exercises)	1	2	3	3	3^a^	3^a^
Static (number of exercises)	1	1	2	2	3^a^	3^a^
**Endurance training**						
Duration	3–5 min	3–8 min	3–8 min	5–10 min	6–12 min	7–15 min
Intensity	low (RPE 10–12)	low (RPE 10–12)	moderate (RPE 12–14)	moderate (RPE 12–14)	moderate to high (RPE 12–15)	moderate to high (RPE 12–15)

*RPE* Rate of perceived exertion, *1-RM* 1-Repetition maximum

^a^same balance exercises, but with increased difficulty

In detail, the resistance training starts with the 2-month accommodation phase. This is followed by a period of regular exercise training with continuous progression of loads until the end of four months. Afterwards, sets and repetitions are further intensified during the last two months (intensified exercise training), when intensity aims to reach subjective muscle fatigue.

Additional coordination and balance training are planned as challenging training according to existing literature [21] and are continuously increased by complexity, difficulty and number of exercises. Coordination and balance are exercised statically on the floor or on a balance pad (AIREX Balance-pad, Switzerland) and dynamically on a balance-platform (HUR Smart Balance, HUR, Finland). This is added by endurance training performed on upright-bicycle and recumbent bicycle ergometers (HUR-Lode ergometers, HUR, Finland). This training will also be performed in a progressive manner regarding intensity and duration.

All training adaptations are communicated by the trainers and depend on the individual´s performance and previous progression. The subjective evaluation and progression of the intensity (resistance training and endurance training) is carried out and monitored throughout the whole training via the BORG scale and rate of perceived exertion (RPE) [25] (Table 1). The training is discontinued in case of safety issues or on participant´s request.

Before each training session, the participants’ state of health is queried and the training schedule is discussed. Training in small groups is intended to additionally foster the motivation to participate in the training on a regular basis. Adherence of the study participants is continuously documented by the exercise instructors during the intervention period. No additional home-based training sessions (exercises performed in the subject´s apartment or room) nor any other non-exercise components are planned. However, in case of any unpredictable short-term training cancellations due to lockdowns during the Corona pandemic, participants will be provided with an alternative home-training programme (i.e. brochure with functional exercises), if necessary. Our study protocol is in line with the Consensus on Exercise Reporting Template (CERT) [27].

#### Usual care

Study participants in the control senior care facilities receive usual care and will not be involved in any aspects of the bestform-training. Instead, the participants are offered 1–2 information meetings by trained study personnel or information brochures on the topic of “Healthy Ageing” during the six months study period.

### Outcome measures

Outcome measures are done at three time-points: baseline, after three months and after six months (end of intervention). Follow-up data will be collected by questionnaires after 18 and 30 months (Table 2).

**Table 2:**
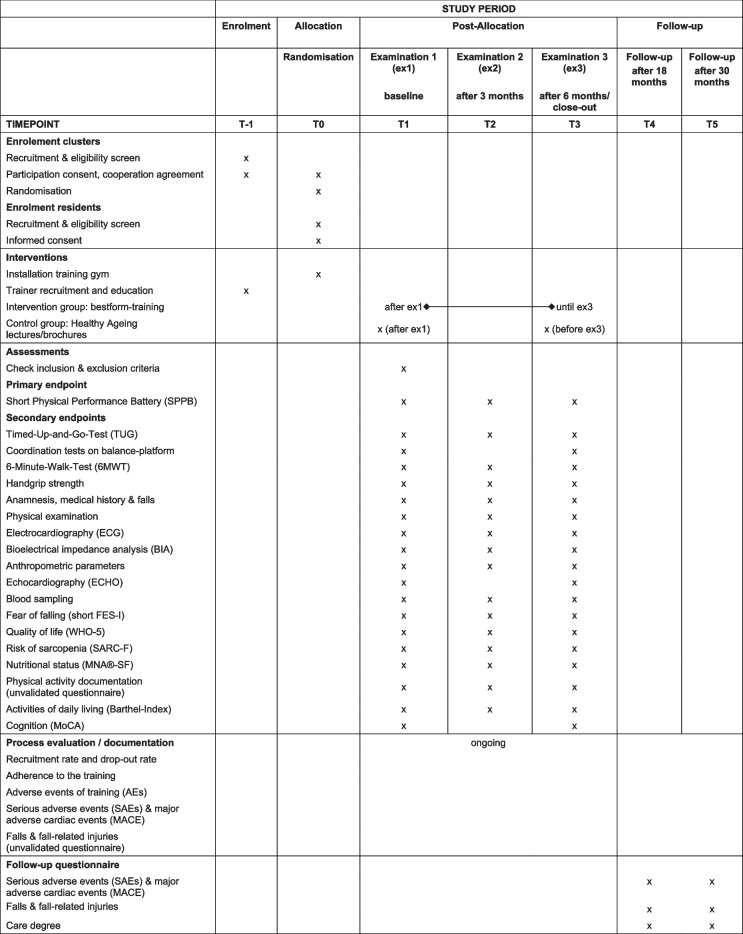
Enrolment, interventions and assessments in the bestform-trial (SPIRIT-Overview)

*MNA®-SF* Mini Nutritional Assessment—Short Form, *MoCA* Montreal Cognitive Assessment, *SARC-F* Screening tool for sarcopenia (questionnaire), *Short FES-I* Short Falls- Efficacy-Scale—International, *WHO-5* WHO-5 Well-Being Index

### Primary outcome

Primary outcome is the change of physical function between baseline and after six months of intervention, measured as the Short Physical Performance Battery (SPPB), a summary performance score out of three tests (standing balance test, gait speed test, and Chair-Stand-Test). For each participant the difference between six months to baseline will be used for analysis.

### Secondary outcomes

Furthermore, secondary outcomes are:

- Change in SPPB after 3 months as compared to baseline
- Change in balance ability, mobility and risk of falling after 3 and 6 months as compared to baseline, measured by the Timed-Up-and-Go-Test and by stability tests on a balance platform
- Change of the 6-Minute-Walk-Test after 3 and 6 months as compared to baseline
- Change in handgrip strength after 3 and 6 months as compared to baseline
- Mortality and rate of hospitalization over 6 months
- Number of falls and fall-related injuries over 6 months, assessed by questionnaire
- Change in fear of falling (short FES-I), quality of life (WHO-5), nutritional status (MNA®-SF), risk screening for sarcopenia (SARC-F), physical activity and activities of daily living (Barthel-Index) recorded by questionnaires after 3 and 6 months as compared to baseline
- Change in fat free mass after 3 and 6 months as compared to baseline, measured by bioelectrical impedance analysis
- Change in calf and upper arm circumference after 3 and 6 months as compared to baseline
- Change in diastolic and systolic left ventricular myocardial function after 6 months as compared to baseline
- Change in proBNP after 3 and 6 months as compared to baseline
- Change in cognition after 6 months as compared to baseline measured by the MoCA

Further secondary endpoints are recruitment rate, adherence, drop-out and lost-to-follow-up rate as well as follow-up evaluation of SAEs, MACE, falls and fall-related injuries, and care degree after 18 and 30 months (Table 2).

### Examinations and assessments

The following examinations are planned at baseline (ex1), after three months (ex2) and after six months (ex3) of the intervention on an individual basis. Echocardiography, the coordination test on a balance platform, and the Montreal Cognitive Assessment (MoCA) [28] will only be assessed at ex1 and ex3. Questionnaires are conducted by interview by trained study staff.

A follow-up questionnaire (Serious adverse events (SAEs), Major adverse cardiac events (MACE) [29], falls, fall-related injuries, care degree) is planned after 18 and 30 months.

#### Anthropometric parameters

The assessment of anthropometric parameters includes weight, height, calf and upper arm circumference as well as body composition, e.g. skeletal muscle mass, measured by bioelectrical impedance analysis (BIA) (Tanita MC-780 MA S; Tanita Europe B.V., Netherlands).

### Assessment for primary endpoint—Short physical performance battery

The Short Physical Performance Battery (SPPB) [30, 31] is based on the tests listed below. For each test 0 to 4 points will be given depending on the performance and of these a SPPB summary score is calculated (0 to 12 points). The higher the score the higher the physical performance.

#### Standing balance test

The standing balance test is a coordination and balance test consisting of three exercises. Duration for each test is 10 s: 1.) Side-by-side position, 2.) Semi-tandem position and 3.) Full-tandem position. Difficulty of the tests is increasing from 1 to 3. Time is documented in all positions until 10 s are completed or the test is prematurely ended. For the first and easiest test to perform (side-by-side position), participants position their feet side by side (parallel in full contact). If the participant is not able to hold the position for 10 s, the complete test is ended and no further tests are started resulting in a standing balance test score of 0 points. If the participant is able to complete the first test, the semi-tandem position is performed next. Also, if the participant is not able to perform the semi-tandem position for 10 s, no further balance tests are started. If the semi-tandem test is successfully completed, then the full-tandem position is conducted.

#### 4 m-walking-test

The 4 m-Walking-test assesses gait speed [32]. The test starts from a standing position and ends when one foot has completely passed the finish line. Participants should perform the test in their habitual gait speed. The faster result of two attempts is used for calculation of a point score (1 to 4 points). If participants are unable to perform the 4 m-Walking-Test, the score will be 0 points. The use of any walking aids (canes, walkers or crutches) is documented.

#### Chair-stand-test

For the Chair-Stand-Test (CST), participants completely rise from a chair for five times as fast as they can. Arms are folded in front of the upper part of the body throughout the whole performance. The test starts with first rising from chair and ends with the final standing position at the end of the fifth repetition. If the participant is not able to rise without assistance of his/her arms or the time needed exceeds 2 min, the CST score is 0 points [33].

### Assessments for secondary endpoints

#### Timed-up-and-go-test

The Timed-Up-and-Go-Test (TUG) is a functional test to uncover gait disorders (muscle function) [34, 35]. The participant sits on a chair. After a start commando, the participant tries to stand up, goes three meters, turns around and sits back down on the chair. The required time is documented in seconds. The use of an assistive device (e.g. cane or walker) is allowed and is documented.

#### Coordination tests on a balance platform

The coordination test is based on quantitative posturography on a force platform (HUR Smart balance, HUR, Finland) and consists of a static and a dynamic exercise. For the static tests, participants remain motionless in upright standing position (heels 2 cm apart, toes pointing outwards as indicated on the platform, 30° angle) with open eyes for 30 s. Subsequently, the same test is performed with eyes closed. Thereafter, the test is repeated on an instable ground (foam mat), with eyes open as well as with eyes closed.

The dynamic test (Limits of stability test) is performed with the feet in parallel position in a hip wide stance (indicated on the platform). The participants have to bend as far as possible forward, backward, left and right without moving or lifting the feet off the platform. Quantitative measurement of postural sway can be used to predict falls in older people [36].

#### 6-Minute-walk-test

To assess the cardio-pulmonary capacity, a 6-Minute-Walk-Test (6MWT) is performed [37, 38]. For this, the participants are asked to walk for 6 min on a 30 m course at a self-paced speed but as rapid and safe as possible. The distance walked within 6 min is documented for each participant. The use of an assistive device (e.g. cane or walker) is allowed and documented.

#### Grip strength

Low grip strength serves as an indicator for an augmented mortality [39]. Grip strength is measured with a hydraulic hand dynamometer (JAMAR, Patterson Medical, USA) in a sitting position. Participants are holding their forearm perpendicular to the upper arm and squeeze the handle of the dynamometer as hard as possible. The test is performed twice and the maximal strength is documented.

#### Fear of falling—Short falls-efficacy-scale – international

To assess the fear of falling, the short form of the Falls-Efficacy-Scale—International (short FES-I) is used. The international questionnaire has been validated for the German language and provides information on level of concern about falls for seven typical activities of daily living with four answer options (1 = not at all concerned to 4 = very concerned) [40].

#### Quality of life – WHO-5 well-being index

Mental and physical health are assessed by the WHO-5 Well-Being Index (WHO-5). The WHO-5 questionnaire comprises five statements to assess subjective mental well-being over the past two weeks. Answer categories are “At no time = 0 point” to “All of the time = 5 points “. The scores of all questions will be summarised and the score then multiplied by 4. The final score ranges from 0 (lowest well-being index) to 100 (highest well-being index) [41].

#### Sarcopenia – SARC-F-questionnaire

The SARC-F-questionnaire is a valid tool for identifying individuals at risk for adverse outcomes from sarcopenia. The SARC-F-questionnaire comprises five items: Strength, assistance with walking, to rise from a chair, to climb stairs and falls with 0 to 2 points for each component resulting in a summary score from 0 to 10 points. A score ≥ 4 points is an indicator for sarcopenia [42]. Further assessments (e.g. grip strength, chair stand test, BIA and SPPB) can be used to quantify sarcopenia, according to official recommendations [8].

#### Nutritional status—Mini nutritional assessment

People over 65 years are often at risk of malnutrition, a condition which also promotes sarcopenia and frailty. To assess the nutritional status the short form of the Mini Nutritional Assessment (MNA®-SF, https://www.mna-elderly.com), a well validated screening tool in many care settings, is used. The instrument comprises six questions to screen for the risk of malnutrition (12–14 points: normal nutritional status; 8–11 points: at risk of malnutrition; 0–7 points: malnourished) [43].

#### Physical activity

Current physical activity is assessed regarding regular current type of activity e.g. walking, bicycling, gymnastics, swimming, duration performed per week and accompanying intensity. The latter is defined by Metabolic Equivalent Tasks (MET) according to Ainsworth et al. [44].

Moreover, former physical activity during lifetime will be assessed according to age and type of sport performed as well as weekly exercise sessions (Additional file 1: Supplement).

#### Activities of daily living

Activities of daily living (ADL) is assessed by the Barthel-Index, which includes 10 questions to assess function and to detect problems in performing activities of daily living, as well as the degree of assistance required with scores from 0 points (difficulties in ADL) to 10 points (no difficulties) with a total score of 100 points. The questions comprise activities such as feeding, personal toileting, bathing, dressing/undressing or control of bladder and bowel. A higher score is associated with a greater degree of independence [45].

#### Cognition test – Montreal cognitive assessment

To assess signs of dementia, the Montreal Cognitive Assessment (MoCA) is to be applied. The 30-points test assesses several cognitive domains including short-term memory, visuospatial abilities, executive functions, attention, concentration and working memory, language and orientation to time and place. The score ranges from 0 to 30 points with lower scores indicating cognitive impairments [46].

MoCA is also used to evaluate the validity of questionnaires. If MoCA score is below 10 points, all other questionnaires will not be considered for evaluation.

### Adverse and serious adverse events

The process evaluation comprises the documentation of recruitment rate and drop-out as well as lost-to-follow-up rate, the documentation of adherence to the training, and the documentation of adverse events (AEs) due to training (complaints while and immediately after training) and serious adverse events (SAEs).

AEs are defined as: Clinical events that occur during the training session or may possibly be related to the training intervention including accidents that occur during the training session or may possibly be related to the training intervention.

SAEs are defined as death, life-threatening events, events that lead to permanent damages or invalidity, or events that are followed by over-night hospitalisation.

AEs due to training and SAEs that occur during the study period irrespective of group assignment are documented in safety forms and transferred to a safety data base. In case of an SAE the principal investigator is directly informed and the procedure on further participation of the study determined e.g. study end, adaptations of the training, temporary or complete cessation of the training.

Throughout the course of the bestform trial, SAEs can be differentiated according to cardiovascular events and analysed separately. MACE are cardiovascular death, non-fatal stroke, and non-fatal myocardial infarction (3-point MACE). MACE and all-cause mortality will be documented during the study and the follow-up phase.

A questionnaire for falls documentation is used at the examinations ex1-ex3, in which the falls and fall-related injuries of the participants are recorded (Additional file 1: Supplement). A falls diary is not applied. Additional documentation is carried out by staff of the senior care facilities or by the trainers in the intervention care facilities (Table 2).

All participants are followed until end of study. A list of SAEs is provided to the responsive Ethics Committee after the end of the study.

### Data monitoring

Monitoring of the study data is performed by the Münchner Studienzentrum (MSZ), Munich, Germany, an independent institution experienced in the monitoring of clinical studies in accordance with standard operating procedures of the MSZ to ensure patients´ safety and integrity of the clinical data, e.g. primary outcome measure and adherence to the study protocol. Monitors have at all times access to all essential documents of the study. All study information is provided to the monitoring personnel during visits. Monitoring procedures are predefined in a study-specific monitoring plan. Furthermore, at the end of the clinical study, a close-out visit is planned to address all activities required for adequate study finalisation.

### Blinding

The primary endpoint is assessed in a blinded manner by independent test personnel. Study participants and physicians, who conduct medical examinations are not informed about the group allocation of the study participants. Secondary endpoints are also partially assessed by unblinded personnel.

### Statistical analysis

#### Sample size calculation – Number of participants

For the primary analysis, mean values in changes of the SPPB score are compared between study groups accounting for the cluster-randomised design. We hypothesise that the intervention group could present at least a difference in mean changes of one point compared to the control group, which is considered as clinically relevant [47, 48]. A within group standard deviation of 2.5 points is assumed [49, 50], translating to an effect size (Cohen’s d) of d = 0.4.

Under these assumptions, 100 participants are needed per group in an individually randomised trial (significance level of 5%, power of 80%, two-sided test). Assuming a mean cluster size of 25 participants and an intra-cluster-correlation (ICC) of 0.025 [51, 52], this sample size has to be multiplied by a Design Effect (DE) of 1.6 to account for the cluster-randomised setting [53], resulting in a sample size of 160 participants per group. As we assume that about 20% of the included individuals will not provide data at the six months assessment, the sample size is increased to 400 individuals in total. To achieve this  ≥ 20 senior care facilities is planned to be recruited.

For the primary analysis comparing the mean values of changes in the SPPB score between the study groups, a mixed effects linear regression model with fixed effect for study group and baseline SPPB score and random cluster effects (senior residences) will be fitted to the data in order to account for within cluster correlation introduced by the cluster-randomised design [54]. The estimated mean difference between the study groups will be represented by the estimated regression coefficient for study group. The null hypothesis of no group difference will be tested two-sided on a significance level of α = 0.05 and a 95% confidence interval for the difference between group mean values will be estimated and presented. Means and standard deviations for both groups will be shown.

Secondary endpoints will be analysed in an exploratory manner. For continuous data, observed means and standard deviations will be presented for both study groups and tests for group differences will be performed as described for the primary analysis. Absolute and relative frequencies for both groups will be presented for binary outcomes. Group comparisons will be conducted by fitting marginal logistic regression models with study group as independent variable to the data accounting for within cluster correlations (generalised estimating equation, GEE models). For count data (e.g. number of falls) corresponding Poisson regression models or negative binomial models will be used. Kaplan–Meier curves will be drawn to illustrate distributions of time-to-event data (time to first MACE) for both study groups. For group comparisons, a Cox regression model with fixed effect for study group and robust standard errors considering the clustering will be fitted to the data. Estimated hazard ratios with corresponding 95% confidence intervals will be presented. Observed adverse events and serious adverse events will be listed and frequencies of adverse events and serious adverse events will be presented. Proportions of individuals with at least one adverse event will be compared between study groups using a logistic GEE model accounting for clustering.

## Discussion

Ageing is often associated with a decrease in physical activity and an increased risk for sarcopenia, frailty and falls [8, 9] as well as other serious diseases, which are also associated with physical inactivity [2]. Therefore, investigating whether a multimodal machine-based strength, coordination and endurance training for old and oldest-old adults living in senior care facilities is safe and has positive effects regarding physical function, risk of falls and various health parameters, is important and will help to improve physical activity programmes in this specific age groups and setting.

According to the United Nations Decade of Healthy Ageing (2021–2030) of the World Health Organization [55], there is a general need for promoting effective physical activity interventions for older adults. However, the optimal intervention is still uncertain. It has been shown that well designed resistance training programmes with appropriate personal instructions are safe for healthy, older adults. It is recommended that these programmes include an individualised, periodized approach with exercises for all major muscle groups [20] and should be offered at least twice weekly involving a combination of upper and lower body exercises with 1–3 sets of 6–12 repetitions [56].

These exercise principles are key elements of our multimodal machine-based bestform-training programme, which is based on the recommendations of the Expert Consensus Guidelines of the International Conference of Frailty and Sarcopenia Research [57]. The training with pneumatic training machines allows an almost infinitely variable adjustment of intensities and thus offers a very individualised approach. The disability-adapted training equipment enables the use in a wide spectrum of individuals with disabilities. Moreover, the three training phases included in the bestform-programme (accommodation, regular exercise training and intensified exercise training) implement progressive training principles according to the current recommendations.

It has been shown that exercise programmes may reduce the risk of falls [10, 58]. For older people living at home, exercise programmes involving balance and functional exercises have shown to reduce the rate of falls by approximately 25% [59, 60]. For older adults living in care facilities, the effect of exercise on rate of falls is uncertain [61]. However, older people living in senior care facilities are in particular need for evidence-based exercise programmes, either by free exercises or machine-based or combined. These may have to exceed those activities usually offered for residents to prevent falls [21].

Particularly progressive machine-based resistance training is rarely offered for the oldest old living in senior care facilities [62]. Reasons are the uncertainty of feasibility and safety, the high cost of equipment and maintenance, and the lack of space or experienced staff. However, there is evidence from our pilot study and others that machine-based training may be feasible and effective, however safety issues have still to be determined. In the machine-based Sunbeam Programme, including 221 participants of 16 residential aged care facilities, the rate of falls was reduced by 55% and the physical performance, measured by the SPPB, was significantly improved without increasing serious adverse events [63]. Our preliminary work confirms the feasibility of the bestform exercise training programme in retirement homes and revealed improvements in the Chair-Stand-Test and the 6-Min-Walk-Test as well as a reduction in fear of falling [22].

In the current bestform trial this approach will be assessed in a far larger setting in a cluster-randomised controlled trial regarding physical function, risk of falling, physical capacity, handgrip strength, number of falls and fall-related injuries, body composition, cardiac function, blood parameters, fear of falling, quality of life, sarcopenia, activities of daily living, and cognition. Aside from efficacy, safety is an important component. If proven safe and effective, the machine-based exercise may be implemented in the routine of senior care facilities and may serve as an approach in addition to free exercises or promotion of daily activities.

A key point of the bestform-training is the supervised training in small groups. Supervision is particular important for older adults, who often have comorbidities, in order to implement an individualised approach and to ensure that the training strain is adequate for the participants, but also safe. A review analysing adherence of older people to training programmes shows a generally higher adherence in supervised programmes [64]. The current World falls guidelines for fall prevention also recommend supervised exercises in care homes [21]. In addition to these aspects, a prescribed functional face-to-face therapy and other more everyday movements (e.g. chair gymnastics) should not be neglected. Furthermore, the group approach strengthens the social exchange and connectedness between the participants. Besides family members, friends also have a great influence on older people’s activity behaviour and group support can encourage regular training [65].

Limitations in this age group of old and very old individuals include a higher morbidity and mortality rate, as well as difficulties performing the training itself, a close monitoring and potential limitations due to cognitive impairments. These issues will also be documented and analysed for future exercise prescriptions.

With the bestform- training programme and its novel setting approach – to apply a machine-based training on site within a senior care facility—we expect a significant mean difference in change in physical function between seniors in the intervention and those living in control care facilities after six months. Particular safety aspects will be assessed in relation to efficacy of the training. The results of the study will contribute to a better knowledge of physical activity concepts for older adults, particularly those living in senior care facilities.

### Supplementary Information

**Additional file 1.** 

## Data Availability

The dataset generated during and/or analysed during the present study will be available from the corresponding author on reasonable request.

## Abbreviations

ADL: Activities of daily living
AEs: Adverse events
BIA: Bioelectrical impedance analysis
CERT: Consensus on exercise reporting template
CONSORT: Consolidated standards of reporting trials
CST: Chair-Stand-Test
DE: Design effect
EA: Easy access
ECG: Electrocardiography
ECHO: Echocardiography
FES-I: Falls-efficacy-scale – international
GEE: Generalised estimating equation
ICC: Intra-cluster correlation
MACE: Major adverse cardiac events
MNA®-SF: Mini nutritional assessment - short form
MoCA: Montreal cognitive assessment
1-RM: 1-Repetition maximum
RPE: Rate of perceived exertion
SAEs: Serious adverse events
SARC-F: Screening tool for sarcopenia
SPIRIT: Standard protocol items: recommendations for interventional trials
SPPB: Short physical performance battery
TUG: Timed-up-and-go-test
WHO-5: WHO-5 (Well-Being-Index)
