# Supplementary material for: Study protocol of a cluster-randomised controlled trial assessing a multimodal machine-based exercise training programme in senior care facilities over 6 months – the bestform study (best function of range of motion)
Source: BMC Geriatr. 2023 Aug 22;23:505. doi: 10.1186/s12877-023-04176-7 (PMC10463394; doi:10.1186/s12877-023-04176-7)
Supplement: Supplementary file 1 — Additional file 1. [file 12877_2023_4176_MOESM1_ESM.zip › Falls_CRF.docx]

| **Falls-CRF**  (page 1 of 2) | **Visit t-1** | **Filled in by:**  _________________________________ |
| --- | --- | --- |
| **Senior Care Facility**    | **Participants-ID**   | **Date**    /   /    |
| **How many times did you fall within the last 12 months** *(unintentionally/involuntarily on the floor)*  I don’t know . ❑ Not at all ❑  Once ❑ Two times or more ❑  **If yes, with injury?**  I don’t know . ❑ Not at all ❑  Once ❑ Two times or more ❑  **Injuries:**  ❑ Bruise ❑ Abrasion ❑ Laceration ❑ Concussion ❑ Fracture ❑ other Injuries    **How many times did you fall within the last 3 months** *(unintentionally/involuntarily on the floor)*  I don’t know . ❑ Not at all ❑  Once ❑ Two times or more ❑  **If yes, with injury?**  I don’t know . ❑ Not at all ❑  Once ❑ Two times or more ❑  **Injuries:**  ❑ Bruise ❑ Abrasion ❑ Laceration ❑ Concussion ❑ Fracture ❑ other Injuries  **Do you have a fear of falling?**  ❑ Not at all ❑ Sometimes ❑ more often ❑ a lot ❑ n. s. | | |
