# Supplementary material for: Study protocol of a cluster-randomised controlled trial assessing a multimodal machine-based exercise training programme in senior care facilities over 6 months – the bestform study (best function of range of motion)
Source: BMC Geriatr. 2023 Aug 22;23:505. doi: 10.1186/s12877-023-04176-7 (PMC10463394; doi:10.1186/s12877-023-04176-7)
Supplement: Supplementary file 1 — Additional file 1. [file 12877_2023_4176_MOESM1_ESM.zip › Physical Activity CRF.docx]

| **Physical Activity CRF** (page 1 of 1) | **Visit t-1** | **Filled in by:**  _________________________________ |
| --- | --- | --- |
| **Senior Care Facility**    | **Participants-ID**  | **Date**    /   /    |
| **Current physical activity:** ❑ yes ❑ no ❑ n.s.   \|  \| **Num-ber of days** \| **Min/**  **Day** \| **Total duration**  **(h/ week)** \| **Intensity**  low / moderate  / vigorous (MET) \| **Total-**  **MET** \| **Total-calories** \| \| --- \| --- \| --- \| --- \| --- \| --- \| --- \| \| Walking \|  \|  \|  \| L M V  3 3,5 4 MET \|  \|  \| \| Bicycling \|  \|  \|  \| L M V  4 5,5 6 MET \|  \|  \| \| Gymnastics \|  \|  \|  \| L M V  Normal 4 MET \|  \|  \| \| Swimming \|  \|  \|  \| L M V  Normal 6 MET \|  \|  \| \| *1  ________________ \|  \|  \|  \| L M V \|  \|  \| \| *2  ________________ \|  \|  \|  \| L M V \|  \|  \| \| Bestform-Training \|  \|  \|  \| L M V  Normal 5,5 MET \|  \|  \| \| Bestform-Home-Training [only in case of corona lockdowns] \|  \|  \|  \| L M V  Normal 4 MET \|  \|  \| \| Total-Activity per week \|  \|  \|  \|  \|  \|  \| \|  \|  \|  \|  \|  \|  \|  \|   ^*1 and *2^: please enter here other sports that have been carried out regularly and have them evaluated with regard to the performance intensity.  **Activity Level (MET x h/week)** = Duration of activity (h/week) x intensity (MET)  **Power consumption (in kcal/week)** = Duration (h/week) x MET x Bodyweight (in kg)  **Have you exercised regularly in the past?**  ❑ yes ❑ no ❑ n.s.   \| **Period of time** \| **What kind of sport?** \| **Hours/ week** \| \| --- \| --- \| --- \| \| Until age of 20 \|  \|  \| \| Age 20-50 \|  \|  \| \| Age 50-65 \|  \|  \| \| Over age of 65 \|  \|  \| \|  \| \| \| | | |
